# Isolation and characterization of two canine melanoma cell lines: new models for comparative oncology

**DOI:** 10.1186/s12885-018-5114-y

**Published:** 2018-12-04

**Authors:** Zacharie Segaoula, Aline Primot, Frederic Lepretre, Benoit Hedan, Emmanuel Bouchaert, Kevin Minier, Laurent Marescaux, François Serres, Sylvie Galiègue-Zouitina, Catherine André, Bruno Quesnel, Xavier Thuru, Dominique Tierny

**Affiliations:** 1 University of Lille, Inserm, CHU Lille, UMR-S 1172 - JPArc - Jean-Pierre AUBERT Research Centre of Neuroscience and Cancer, F-59000 Lille, France; 2 OCR (Oncovet Clinical Research), SIRIC ONCOLille, Parc Eurasante, Rue du Dr Alexandre Yersin, F-59120 Loos, France; 3 CNRS-University of Rennes 1, UMR 6290, Institute of Genetique and Development of Rennes, Faculty of Medicine, SFR Biosit, Rennes, France; 4 Functional Genomic Platform, Univ. Lille, F-59000 Lille, France; 5 Oncovet Cancer Centre, Avenue Paul Langevin, 59650 Villeneuve d’Ascq, France

**Keywords:** Melanoma, Cell lines, Comparative oncology, Preclinical models, Cancer, Dog

## Abstract

**Background:**

Metastatic melanoma is one of the most aggressive forms of cancer in humans. Among its types, mucosal melanomas represent one of the most highly metastatic and aggressive forms, with a very poor prognosis. Because they are rare in Caucasian individuals, unlike cutaneous melanomas, there has been fewer epidemiological, clinical and genetic evaluation of mucosal melanomas. Moreover, the lack of predictive models fully reproducing the pathogenesis and molecular alterations of mucosal melanoma makes its treatment challenging. Interestingly, dogs are frequently affected by melanomas of the oral cavity that are characterized, as their human counterparts, by focal infiltration, recurrence, and metastasis to regional lymph nodes, lungs and other organs. In dogs, some particular breeds are at high risk, suggesting a specific genetic background and strong genetic drivers. Altogether, the striking homologies in clinical presentation, histopathological features, and overall biology between human and canine mucosal melanomas make dogs invaluable natural models with which to investigate tumor development, including tumor ætiology, and develop tailored treatments.

**Methods:**

We developed and characterized two canine oral melanoma cell lines from tumors isolated from dog patients with distinct clinical profiles; with and without lung metastases. The cells were characterized using immunohistochemistry, pharmacology and genetic studies.

**Results:**

We have developed and immunohistochemically, genetically, and pharmacologically characterized. Two cell lines (*Ocr_OCMM1X* & *Ocr_OCMM2X*) were produced through mouse xenografts originating from two clinically contrasting melanomas of the oral cavity. Their exhaustive characterization showed two distinct biological and genetic profiles that are potentially linked to the stage of malignancy at the time of diagnosis and sample collection of each melanoma case. These cell lines thus constitute relevant tools with which to perform genetic and drug screening analyses for a better understanding of mucosal melanomas in dogs and humans.

**Conclusions:**

The aim of this study was to establish and characterize xenograft-derived canine melanoma cell lines with different morphologies, genetic features and pharmacological sensitivities that constitute good predictive models for comparative oncology. These cell lines are relevant tools to advance the use of canine mucosal melanomas as natural models for the benefit of both veterinary and human medicine.

**Electronic supplementary material:**

The online version of this article (10.1186/s12885-018-5114-y) contains supplementary material, which is available to authorized users.

## Introduction

In humans, melanoma is one of the most aggressive types of cancer. Whereas cutaneous forms represent the most common cases, melanoma of the oral cavity is rare but very aggressive, highly metastatic [1] and associated with frequent relapse and poor outcomes [2]. These forms mainly arise in the sino-nasal cavity (more than 73% of cases) and are highly immunogenic tumors contributing to an immune anti-tumor reaction that can lead to tumor escape and resistance to most standard treatment protocols [3]. Mihajlovic and colleagues reported in 2012 that most primary mucosal human melanomas clinically evolve in an aggressive manner with 25% five-year survival rates, which are much lower than those of the cutaneous (80.8%) and ocular (74.6%) forms [4]. To date, conventional chemotherapy has played an uncertain role in the treatment of these tumors, while targeted treatments, based on Braf inhibitors are mainly used on the tumors harbouring the mutated isoform of this protein, leaving a large proportion of untreatable forms.

In dogs, although local and regional control of oral canine malignant melanomas (CMMs) using surgery followed by adjuvant radiation may be successful in preventing tumors from spreading, the preventive use of poly-chemotherapeutic protocols using cytotoxic drugs has previously been considered non-productive, with no real benefit nor any extension of survival time [5–8].

CMMs are one of the most frequently diagnosed malignancies of the oral cavity. These cancers account for 7% of all malignant tumors in dogs, with 160,000 cases reported worldwide every year [9, 10] and are the most frequent form of melanoma in canines (62%) in a set of 1652 melanomas and the most aggressive, with a median survival rate of 200 days vs. 474 days for the cutaneous type [9]. These non-UV-dependent malignancies are some of the most aggressive metastasizing tumors, with a median post-surgery survival rate of 173 days [8, 11]. Based on available genetic tools, medical monitoring and its pre-clinical trial potential, CMM appears as a reliable model for human cancers, especially melanomas [12, 13, 17].

**Fig. 1:**
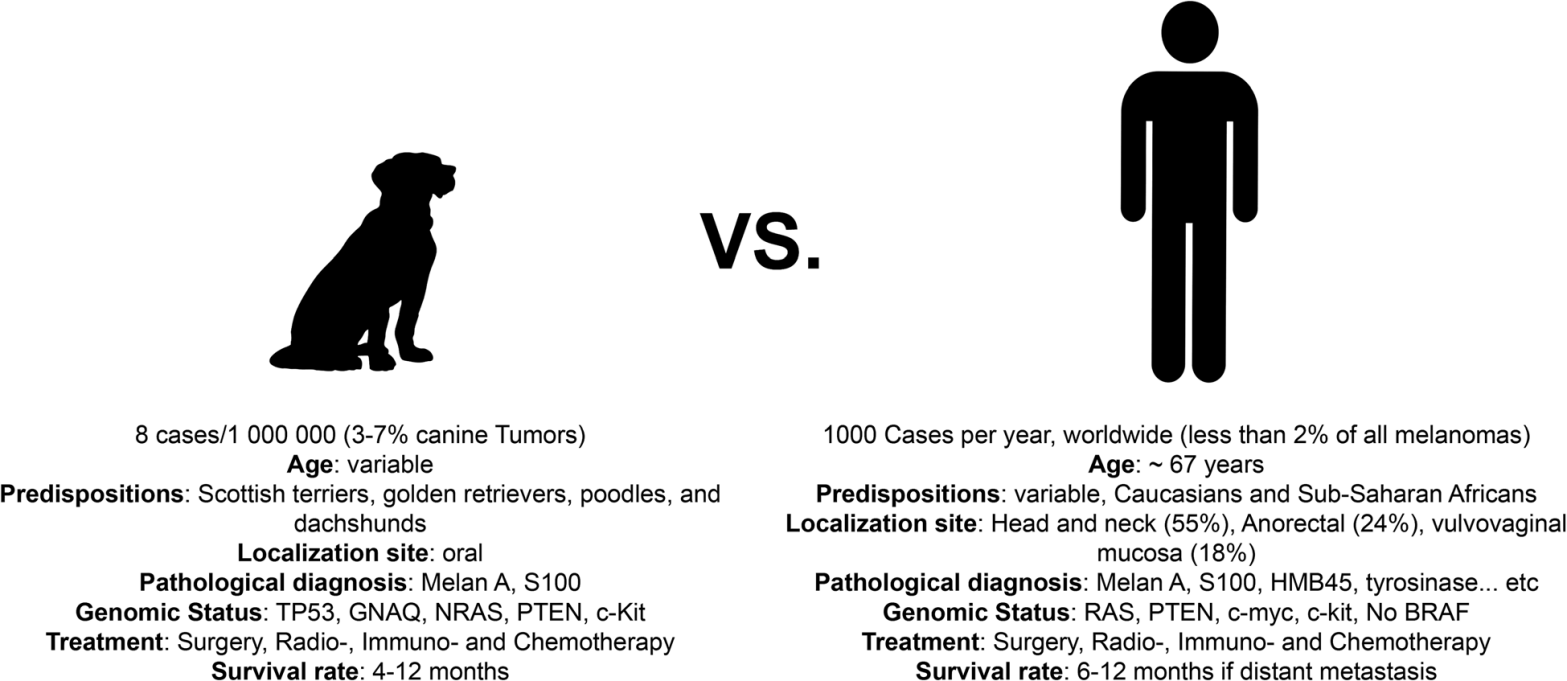
Similarities between canine and human mucosal melanomas (adapted from [42] and [43])

Human and canine oral melanomas share multiple morphological, cytogenetic, molecular and signaling pathway similarities [14–16] (Fig. 1). In fact, dogs share the same environment as humans and are therefore exposed to the same carcinogens that may be involved in tumorigenesis. Even though some breeds are more naturally predisposed to certain types of cancers, largely due to centuries of selective breeding, others develop spontaneous neoplasms that naturally reflect the tumorigenic process, in contrast to experimentally induced mouse models [17]. These neoplasms have also pathologic features, biological behavior, clinical course response and resistance to treatment similar to those of their human counterparts [18]. Indeed, CMMs are generally aggressive, highly metastatic and associated with poor prognoses, but some canine patients with early stage tumors or benign lesions may develop extended survival times [19, 20].

All the analogies reported between CMM and human mucosal melanoma regarding the biology, the clinical behavior and the poor prolonged survival times with existing conventional therapies make investigations in companion animals with spontaneous melanomas attractive comparative models for developing novel, less toxic and more effective treatment strategies for both species. Only a few canine melanoma cell lines have already been reported in the literature. For instance, Inoue et al. established four canine melanoma cell lines (*CMec-1 CMec-2 KMec* and *LMec*) of various origins (subcutaneous, gingival and mucosal) from donors with different stages of development (T3 N1 M0 and T4 N1 M0). Of the three cases, no distant metastases were reported and only morphological characterization was performed [21]. Our study completed and endorsed this work by providing further validation of canine melanoma as models for translational studies.

Here, we established and morphologically, immunohistochemically, pharmacologically and genetically characterized different canine melanoma cell lines. Samples were obtained from two dog patients presenting with spontaneous melanomas with different stage of malignancy. The objective of our project was to investigate the potential aggressiveness and tumorigenesis of these cells in vivo and to identify the common aspects that could be linked to human pathology. The obtained results indicate that the 2 cell lines that we developed are capable of inducing tumor growth in a mouse xenograft model. Additionally, the growth curves vary between the two samples, mirroring the aggressiveness and biology of the tumors from which they originated. Therefore, we presume that both cell lines constitute relevant models for comparative oncology clinical studies and may provide mutual benefits for both canine and human research.

## Material and methods

### Cells

#### Canine melanoma cell lines

**Fig. 2:**
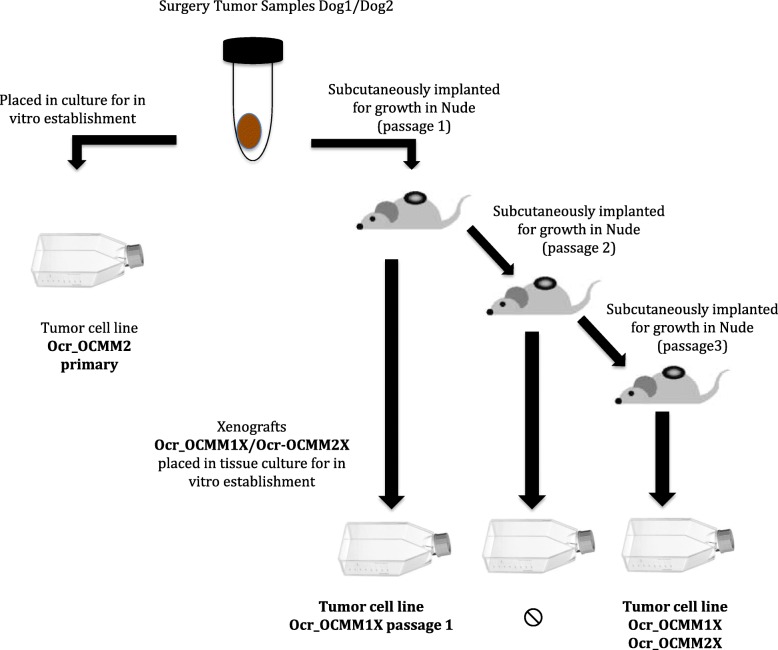
Schematic protocol of new melanoma canine cell lines establishment

Ocr_OCMM2 primary culture was obtained after 12 months cell culture. Ocr_OCMM1X passage 1 was obtained after one xenograft in Nude mice and in vitro cell culture. Ocr_OCMM1X and Ocr_OCMM2X were obtained after 3 xenograft passages followed by in vitro culture during 10 months (Fig. 2).

#### Ethics statement

Dogs were recruited in France from client-owned dogs that were presented at a specialist veterinary clinic (Oncovet Cancer Centre, Villeneuve d’Ascq, France), and informed consent was obtained from pet owners. Tumor material was surgically excised and handled according to the standard of care and under controlled aseptic conditions at the Oncovet Cancer Centre. Animals received, according to their corresponding stage of the disease, the standard chemotherapy and/ or radiotherapy cycles, following the recommendations defined by the European College of Veterinary Oncology.

#### Tumor samples

Melanoma tumor tissues were obtained from a 14-year-old Yorkshire Terrier (TS1, Tumor sample 1) and an 11-year-old German Shepherd (TS2, Tumor sample 2) that presented at the clinic with melanocytic lesions of the oral cavity with and without metastasis to the lungs (Table 1). Two xenograft-derived canine melanoma cell lines (Ocr_OCMM1X and Ocr_OCMM2X) were obtained from these primary tumors and one cell line was established directly from in vitro culture a melanoma tissue from Dog 2 (Ocr_OCMM2 primary) (Fig. 2).

**Table 1:** Clinical, morphological and functional characteristics of the isolated tumor cell lines

Dog			Tumor					Cell line			
Breed	Age	Sex	Primary site	WHO stage	Clinical stage	Histologic Pattern	Distant Metastasis	Name	Origin	Number of passages	PDT (h)
Yorkshire Terrier: Dog_1	14	Female	Mucosal	T2N1M0	III	Polygonal cells	No	Ocr_OCMM1X	Oral CMM	36	59,9 ± 6
								Ocr_OCMM1X passage 1		25	41,8 ± 8
German Sheprherd: Dog_2	11	Female	Gingival	T2N1M1	IV	Spindle and Polygonal Cells	Yes	Ocr_OCMM2 primary	Oral CMM	20	75,1 ± 8
								Ocr_OCMM2X		38	61,6 ± 4

*LN* Lymph Node, *TNM* Tumor Node Metastasis, *PDT* population doubling times

#### Human melanoma cell lines

The human melanoma cell lines A375 and Sk-Mel28 were obtained from the American Type Culture Collection. Cells were grown in a humidified 5% CO_2_ atmosphere at 37 °C in RPMI medium containing Glutamax (Invitrogen) supplemented with 10% fetal bovine serum and 100 μg/ml primocin (Invivogen).

Isolation and cell culture. Primary cells (from Dog 1 and Dog 2 tumor samples) were immediately obtained from the isolated extracts of the digested tissues. Surgically removed oral canine melanoma tissue samples were digested mechanically and enzymatically using 2 mg/mL type II collagenase (Thermo Fisher Scientific, Waltham, MA) for two hours at 37 °C. After complete dissociation, cells were filtered with a 70 μm sterile nylon cell strainer and cultured in complete RPMI 1640 Glutamax growth medium supplemented with 10% heat-inactivated fetal calf serum and antibiotics (penicillin 100 U/mL and streptomycin 0.1 mg/mL) (Thermo Fisher Scientific, Waltham, MA) at a final density of 107 cells per 75 cm2 culture flask for primary culture (Ocr_OCMM2 primary). Then, after tumor growth in Nude mice, the same protocol was applied to isolate cells from the engrafted tumors (Ocr_OCMM1X & Ocr_OCMM2X). Cells have been cultured during 12 consecutive months before genomic analysis. Cells were passaged several times in T75 culture flasks before freezing, with careful attention during trypsinization. No effect of the freezing/ thawing cycles on cell growth was observed.

### In vivo tumorigenesis studies

#### Animals

Severe combined immunodeficient Hairless Outbred (SHO®) Nude mice were purchased from Charles River (Wilmington, MA) and kept under specific-pathogen-free conditions in individual IVS-type cages within the core animal facility of PLETHA (Institute Pasteur de Lille, France). All protocols were approved by the institutional animal care and ethics committee and are in accordance with the European parliament directive (2010/63/EU).

#### First xenografts

Both surgically removed oral CMM tissue samples were injected subcutaneously (s.c.) at the interscapular site of 12-week-old mice (*n* = 5 for each tumor sample, Dog_1 vs. Dog_2). Tumor growth was monitored every 3–4 days. Tumor volume was evaluated using a caliper and then calculated as 0.5 × length × width^2^. Mice were euthanized when their tumors reached 2000 mm^3^ in volume. The tumors were harvested and prepared for cancer cell isolation in T75 culture flasks under sterile conditions (Ocr_OCMM1X passage 1), as previously described [22]. Two additional successive xenograft namely passage 2 and 3 were performed using tumor samples issued from previous passage.

#### Inoculation of melanoma cells

Two million (2 × 10^6^) melanoma cells of each previous xenograft were suspended in 100 μL of Matrigel solution and injected s.c. at the interscapular site of 12-week-old mice (n = 5 for each group injected with previous passage xenograft tumor samples). Tumor growth was monitored as described above. Mice were euthanized when their tumors reached 2000 mm^3^ in volume. Tumors were harvested, and the cells were extracted and cultured in flasks under sterile conditions (xenograft-derived cell line Ocr_OCMM1X passage 1 and Ocr_OCMM2X passage 1).

#### Evaluation of cell growth

Population doubling times (PDT) were evaluated for each cell line (Table 1). Cells (Ocr_OCMM2 primary, Ocr_OCMM1X passage 1, Ocr-OCMM1X and Ocr_OCMM2X) were plated at a density of 1 × 10^5^ cells in 60 mm petri dishes with 4 mL of complete growth medium and then incubated at 37 °C in a controlled humidified atmosphere with 5% CO_2_ for 96 h. Cells were trypsinized (0.25% EDTA) and counted every day in duplicate, and the PDT was calculated using the following formula: PDT = 1/ [3.32(logNH −logNI)/ (t2 − t1)] (where t1 = time in hours when cells were seeded; t2 = time in hours when cells were harvested; NI = cell count when cells were seeded; NH = cell count when cells were harvested) as described by *Rütgen* et al [23].

### Histological characterization

#### Immunohistochemistry

Collected tumor tissues from dogs and xenograft were fixed in 10% buffered formalin solution (Sigma-Aldrich, St Louis, MI) and paraffin embedded for routine pathological analysis. Immunohistochemical staining was carried out on 4- to 6-μm-thick slides using an automated protocol developed for the Discovery XT automated slide staining system (Ventana Medical Systems, Inc*.*). Alongside haematoxylin–eosin staining (HE), the following commercially available antibodies were used for the phenotypical characterization of melanocytes: Melan-A (Thermo Fisher Scientific, Waltham, MA), S-100 protein (Dako, Agilent Technologies, Denmark), Vimentin, and cytokeratin (Biogenex Laboratories, San Ramon, CA) (see Additional file 1). Tumor sections were deparaffinized and incubated for 1 h with the appropriate antibody before incubation with Discovery UltraMap anti-Rabbit (760–4315, Roche, Basel, Switzerland) or anti-mouse horseradish peroxidase (HRP) (760–4313, Roche, Basel, Switzerland) secondary antibodies and the Discovery ChromoMap DAB kit reagents (760–159, Roche, Basel, Switzerland) (Additional file 1).

#### Immunofluorescence analysis

Cells (Ocr_OCMM1X and Ocr_OCMM2X) were grown on pre-coated poly-lysine 4-well chamber slides (Lab-Tek, Nunc®, Roskilde, Denmark) and stained as previously described [22]. The cells were then incubated with monoclonal anti-Melan A or anti-S100 antibodies (Dako, Glostrup, Denmark) for 1 h at room temperature, washed three times, and incubated for 1 h at room temperature with 2 μg/mL donkey anti-rabbit (A-21206, Thermo Fisher Scientific, Waltham, MA) or goat anti-mouse Alexa Fluor 488 (Thermo Fisher Scientific, Waltham, MA) secondary antibodies. Fluorescence microscopic analysis was performed with a Leica DMRB microscope (Leica Microsystems) with a PL Plan-Leica Fluotar 20×/1.00 objective. Photographs were taken with a DFC345FX digital camera and processed with the Leica Application Suite (LAS v 3.7 software) (Leica Microsystems, Wetzlar, Germany), and images were further processed using ImageJ® software.

### Pharmacological characterization

#### MTS cytotoxicity assay

The cells used for MTS cytotoxicity assay, A375, Sk-Mel28, Ocr_OCMM2X and Ocr_OCMM1X, were seeded at 5000 cells/cm^2^ at each passage, with 2 or 3 passages per week. Prior to any treatment, the cells (A375, Sk-Mel28, Ocr_OCMM1X & Ocr_OCMM2X) were seeded in flat bottomed 96-well plates at a density of 2 × 10^3^ cells per well with complete medium. Cells were treated the following day with 1% DMSO vehicle control or with a four-fold serial dilution of chemotherapeutic agent ranging from 24 nM to 400 μM dacarbazine, an alkylating agent (Medac, 500 mg, Teva, Petah Tikva, Israel), 6.1 nM to 100 μM vemurafenib, a BRAF inhibitor (Selleckchem, Houston, TX) and 6.1 nM to 100 μM LY294002, an inhibitor of PI3Kα/δ/β and autophagosome formation (Selleckchem, Houston, TX). To quantify cellular proliferation, MTS assays (Promega, Fitchburg WI) were performed by adding 20 μL of MTS solution to each well and incubating at 37 °C for 1 to 2 h. The optical density of each well was acquired with a spectrophotometric plate reader (FLUOstar Omega, BMG Labtech, Germany) at a wavelength of 490 nm in accordance with the supplier’s instructions. Experiments were performed in 6 replicates. The results for each treatment were reported as the mean of the percent surviving cells ± SEM (standard error of the mean). IC50 values were obtained using GraphPad Prism™ software, version 6.1 (Sun Microsystems, Palo Alto, CA, USA).

#### Western blot analysis

Cells (Ocr_OCMM1X and Ocr_OCMM2X) were seeded in 60 mm petri dishes at a final concentration of 5 × 10^6^ cells per flask 24 h prior to treatment. Either the control solution or 5 μM vemurafenib was applied for 24 and 48 h. Cells were washed twice with PBS and then harvested, and the protein extracts were prepared for Western blotting. Briefly, protein extracts were isolated by lysis of the harvested cell pellets with RIPA buffer (50 mM Tris, pH 7.5, 150 mM NaCl, 1 mM EDTA, 1% NP-40, 0.25% Na-deoxycholate and 1 mM PMSF) supplemented with a protease inhibitor cocktail (Sigma-Aldrich, St-Louis*,* MI). The protein concentration was determined by the Pierce™ BCA protein assay kit (Thermo Fisher Scientific*,* Waltham, MA). Twenty micrograms (20 μg) of protein was subjected to 10% SDS-NuPAGE precast polyacrylamide gel electrophoresis and then transferred to a nitrocellulose membrane (Thermo Fisher Scientific Inc., Waltham, MA). Next, the protein was incubated with antibodies directed against MEK, phosphorylated-MEK (pMEK), AKT, phosphorylated-AKT (Cell Signaling Technology, Danvers, MA) and HSC-70 (Santa Cruz Biotechnology, Santa Cruz, CA), according to the supplier’s instructions (see Additional file 1). Finally, the blots were visualized using the Amersham ECL Prime Western Blotting System on a Las 4000 biomolecular imager and further analyzed by ImageQuant tools (GE Healthcare Europe GmbH, Velizy-Villacoublay, France).

### Molecular characterization

#### DNA extraction

Total tissue and cellular DNA were isolated from each corresponding tumor sample and cell line pellet using the Qiagen DNeasy Kit according to the manufacturer’s instructions (Qiagen, Hilden, Germany). The samples were assessed for quality and quantity by spectrophotometry using a NanoDrop spectrophotometer (Thermo Fisher Scientific, Waltham, MA) and for genomic DNA integrity by agarose gel electrophoresis, showing no significant degradation.

#### Somatic mutations in candidate genes

Genomic DNA sequencing was performed on six genes that frequently harbour recurrent somatic mutations in human melanoma subtypes: *Braf*, *Nras*, *Gna11*, *Gnaq*, *Mitf* and *p53* (Additional file 2). The genes were sequenced in the canine tissue-derived genomic DNA isolated from the Dog_1 primitive tumor, the Dog_1 engrafted tumor and Ocr_OCMM1X cells, as well from the Dog_2 primitive tumor, and the Dog_2 engrafted tumor, and the primary derived and Ocr_OCMM2X cells. Each gene was sequenced from the genomic DNA, with the Sanger method, as previously described [17]. Data were analyzed with DNA Sequencing Analysis software v5.2 (Applied Biosystems). The presence of somatic mutations was assessed by comparing DNA sequences from the sample to the CanFam3 reference dog sequence using Seqscape software v2.5 (Applied Biosystems™, Foster City, CA, USA).

#### Chromosome alterations

An oligo array comparative genomic hybridization (CGH) analysis was carried out on the DNA previously extracted from Dog_1 and Dog_2, using pan-genomic arrays of 60-mer oligonucleotides, comprising a total of 180,000 probes (Canine Genome 4 × 180 K SurePrint G3 Canine CGH Microarray 025522_D_F_20130822; Agilent Technologies, Santa Clara, CA). The arrays were scanned on an Agilent G2505C scanner, the images were quantified using Agilent Feature Extraction software (vA.8.5.1.1), and the acquired images were analyzed using Agilent Genomic Workbench software (V7.0.4.0). The canine genome build CanFam3.1 was used for data analysis. Copy number aberrations (CNA) were identified using aberration detection method 2 (ADM2) segmentation and the R script DNA library. A CNA was called as either a gain or deletion according to the log2 ratio distribution. Probes on chromosomes X or Y were not analyzed because of sex mismatching. Regions of heterozygous gain (amplification) were defined as having a log2 ratio > + 0.25, while regions suggestive of heterozygous loss (deletion) were defined as having a log2 ratio < − 0.25. (The threshold was fixed at 6.0). The array data presented here are publicly accessible through the Gene Expression Omnibus (GEO) Series, accession number GSE88724.

### Statistical analysis

Results are expressed as the mean ± SEM, and statistical analyses were performed using GraphPad Prism® and StatView® 5.0 software (SAS, Cary, NC). Data were analyzed using ANOVA followed by Fisher’s PLSD post hoc test with a significance level of *P* < 0.05 (*p < 0.05; **p < 0.01; ***p < 0.005; ****p < 0.001).

## Results

**Fig. 3:**
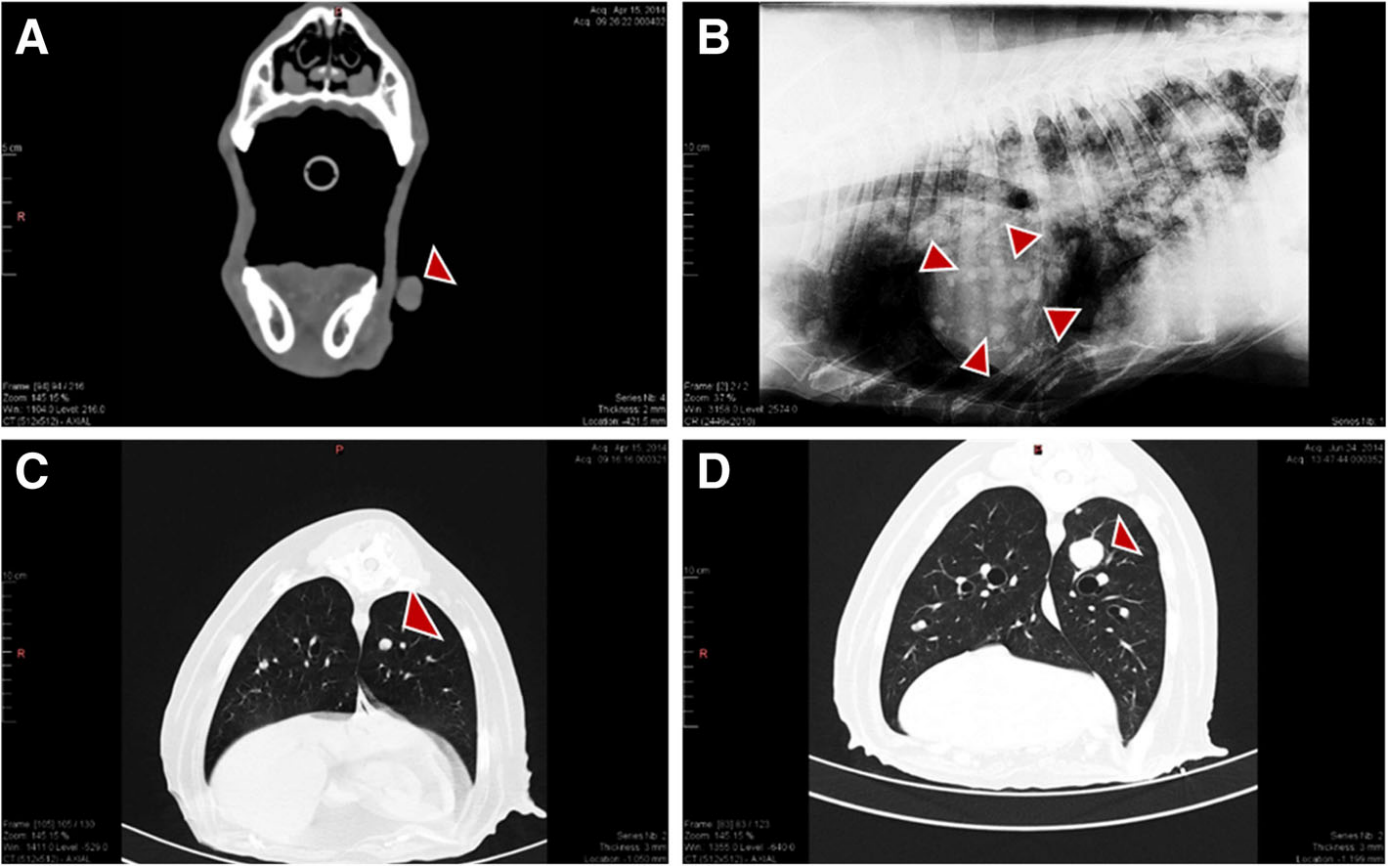
CT-scans showing Dog_2 primary tumor mass in the oral cavity (red arrow head) (**a**) and lateral radiographic projection displaying distant metastasis in the thoracic region (**b**). CT-scans in **c** and **d** show tumor clinical follow-up three and six months after treatment initiation respectively

Melanoma tumor tissues were obtained from a 14-year-old Yorkshire Terrier (Dog_1) and an 11-year-old German Shepherd (Dog_2). The histopathological reports confirmed the diagnosis of malignant melanoma while CT scan of oral cavity and thorax completed the tumor staging. Both tumors were in the oral cavity but presented different WHO stage at the time of diagnosis and samples collection (Table 1). While the first case (Yorkshire terrier, Dog_1) with stage T2N1M0 only had metastases to regional lymph nodes, the second case (German shepherd, Dog_2) with stage T2N1M1 had metastases also in the lungs, which grew rapidly from 3 to 8 mm into 24 mm and invaded the central nervous system six months after surgery (Fig. 3). Unfortunately, due to the euthanasia of Dog_1 three months after diagnosis, with no follow-up CT scan of thorax, the creation of lung metastasis could not be confirmed or excluded.

### Establishment and characterization of canine melanoma cell lines

After a minimum of 5 passages (two months), population doubling times were evaluated, and showed that the primary derived cells from Dog_2 (Ocr_OCMM2_Primary cells) tend to cycle very slowly (75.1 ± 8.0 h), whereas the xenograft-derived cell line Ocr_OCMM2X grows slightly faster (61.6 ± 4.0 h) (Table 1).

The same decrease in the doubling time was observed for the cells derived from Dog_1 (Ocr_OCMM1X passage 1 and Ocr_OCMM1X), with 59.9 ± 6.0 h and 41.8 ± 8.0 h for the first and second passages, respectively.

**Fig. 4:**
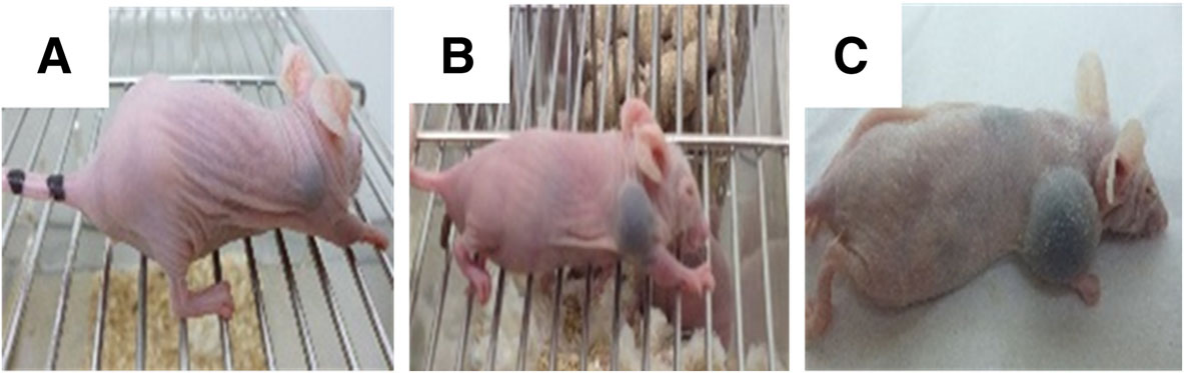
In-situ tumor growth of Ocr_OCMM1X passage 1 cell line: 45 (**a**), 65 (**b**) and 100 (**c**) days post-injection

**Fig. 5:**
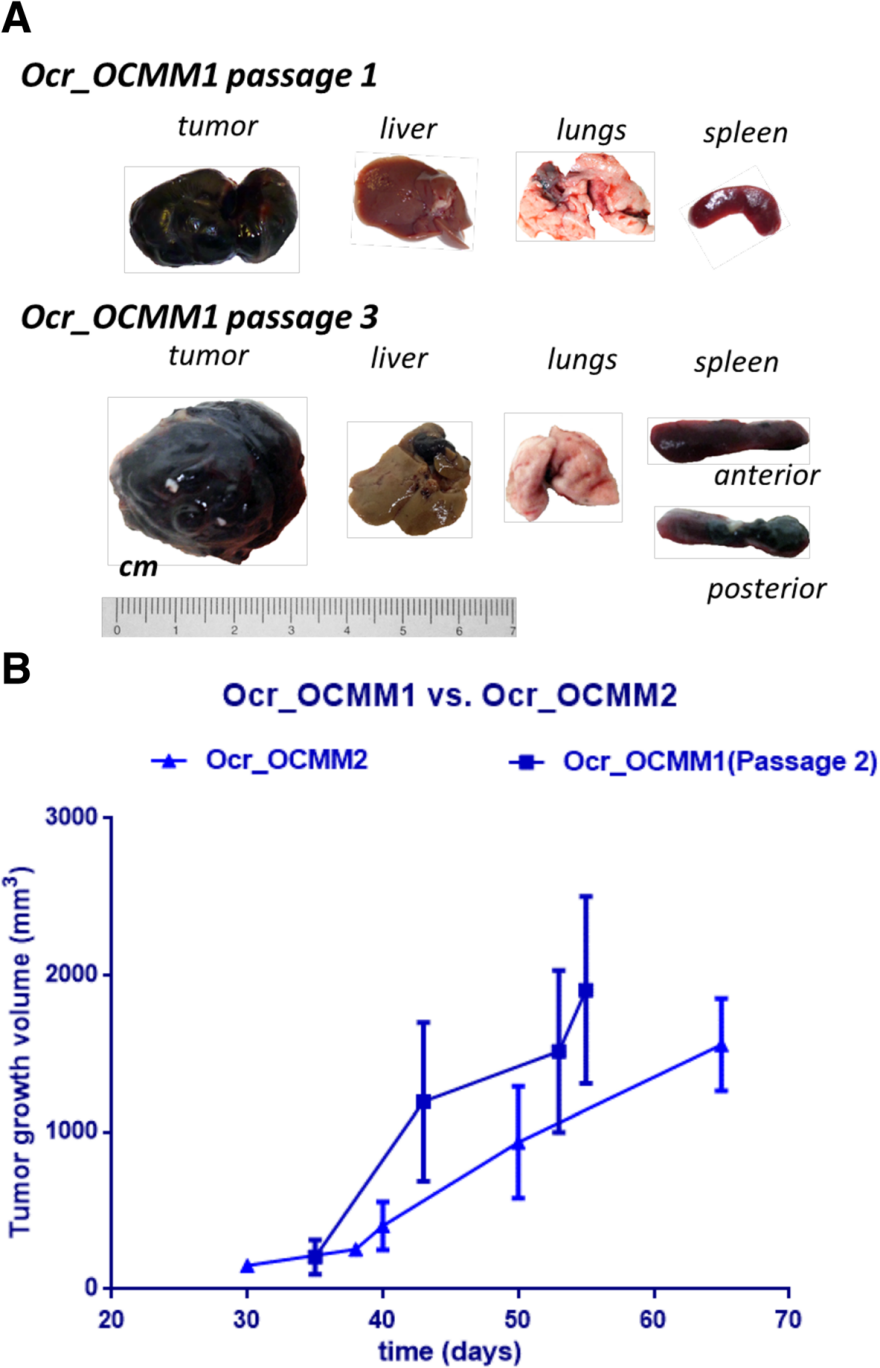
Macroscopic evaluation of necropsy material from Dog_1 (Ocr_OCMM1) engrafted tumors (**a**) and growth curves of Dog_2 (Ocr_OCMM2) (first passage) and Dog_1 (Ocr_OCMM1) (second passage) cell lines (**b**). The figures show that after three passages, metastases were found in the liver, spleen and lungs of the injected mice and tumors grew faster and bigger in both groups

Xenograft-derived cell lines were obtained from tumors in subcutaneously injected immunodeficient mice (Fig. 4). After 45 days, palpable black lumps with volumes of 56.1 ± 21.1 mm^3^ were observed in the group engrafted with the cells isolated from Dog_2’s tumor (TS2) (Fig. 4). Three out of five mice developed tumors that reached 2000 ± 288.7 mm^3^ within 110 days of inoculation during the first passage (data not shown). A rapid onset of the tumor engraftment of TS2 (from Dog_2) compared to TS1 (from Dog_1) during the first passage reflected higher stage of malignancy at the time of diagnosis and samples collection the natural aggressiveness of the tumor. Tumors arising from Dog_2 grew faster, with a median tumor volume of 1600 ± 295 mm^3^ in the period between 50 and 65 days (Fig. 5b). All mice developed tumors, and no metastasis was reported during the first passages (Fig. 5a).

### Morphological and phenotypical characterization

**Fig. 6:**
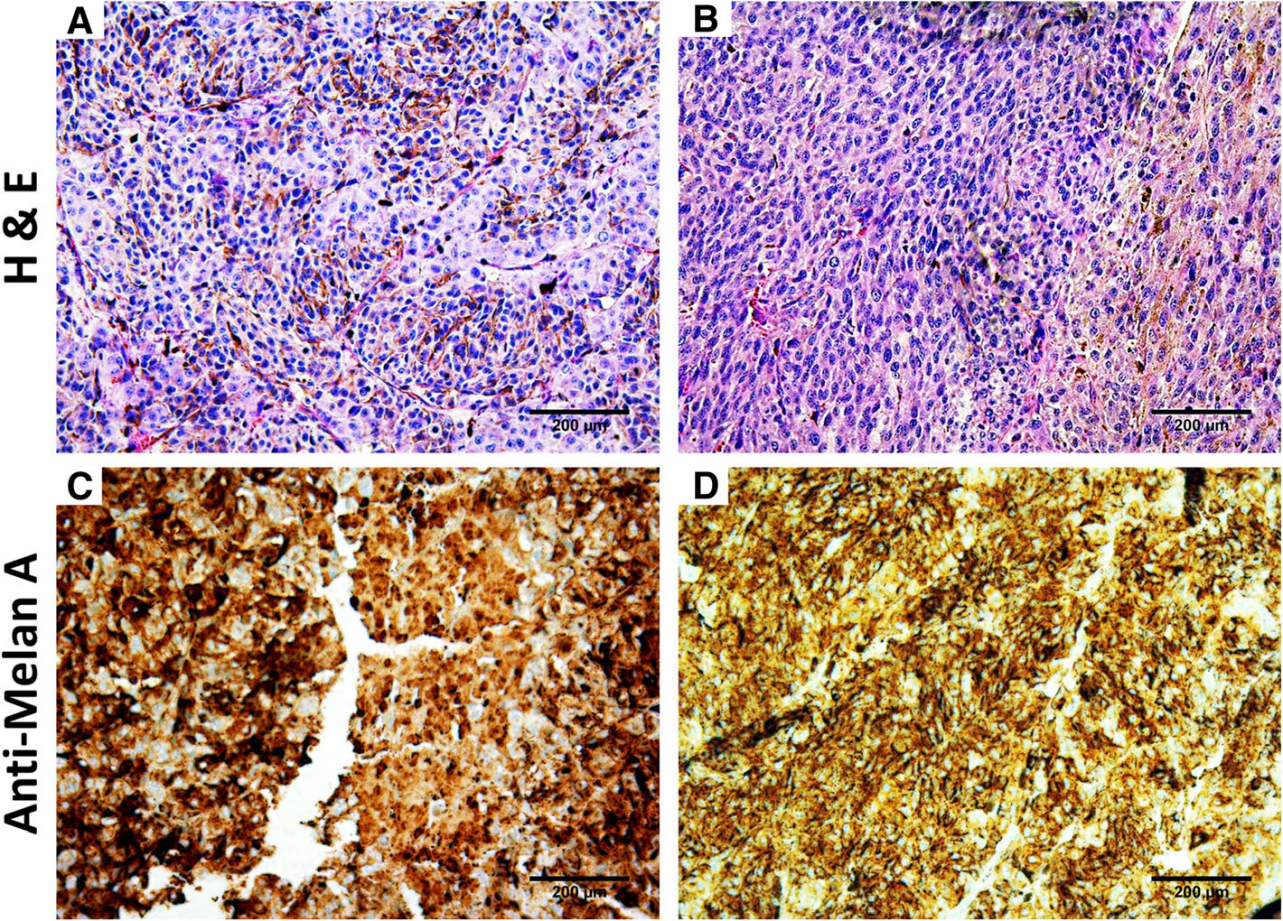
Immunohistochemical analysis of Ocr_OCMM1 (**a, c**) and Ocr_OCMM2 (**b, d**) engrafted tumors. Hematoxilin & Eosin and Melan-A colorations show a large, diffuse and heterogenous staining of the tumor mass tissue. Observation under light microscope (magnification X200)

The engrafted formalin-fixed tumors were histologically characterized by a veterinary pathologist. The cells presented the characteristics of highly cellular melanoma with polymorphic epithelioid, spindloid or polygonal mixed populations (Additional file 3A). Lesions were well delineated, with discrete local invasion. The cells were large (> 30 μm) and presented abundant fibrillary, acidophilic cytoplasm with large, distinct, clear vacuoles, round nuclei with coarse chromatin and small prominent single nucleoli (Additional file 3A). Cytonuclear analysis showed moderate cytological atypia of melanocytes (anisocytosis and anisokaryosis) with 3 mitoses per high-power field (Fig. 6a, b, Additional file 3A). Brown dark-pigmented melanosomes were also found. These observations confirmed the diagnosis of melanoma (Additional file 3A).

**Fig. 7:**
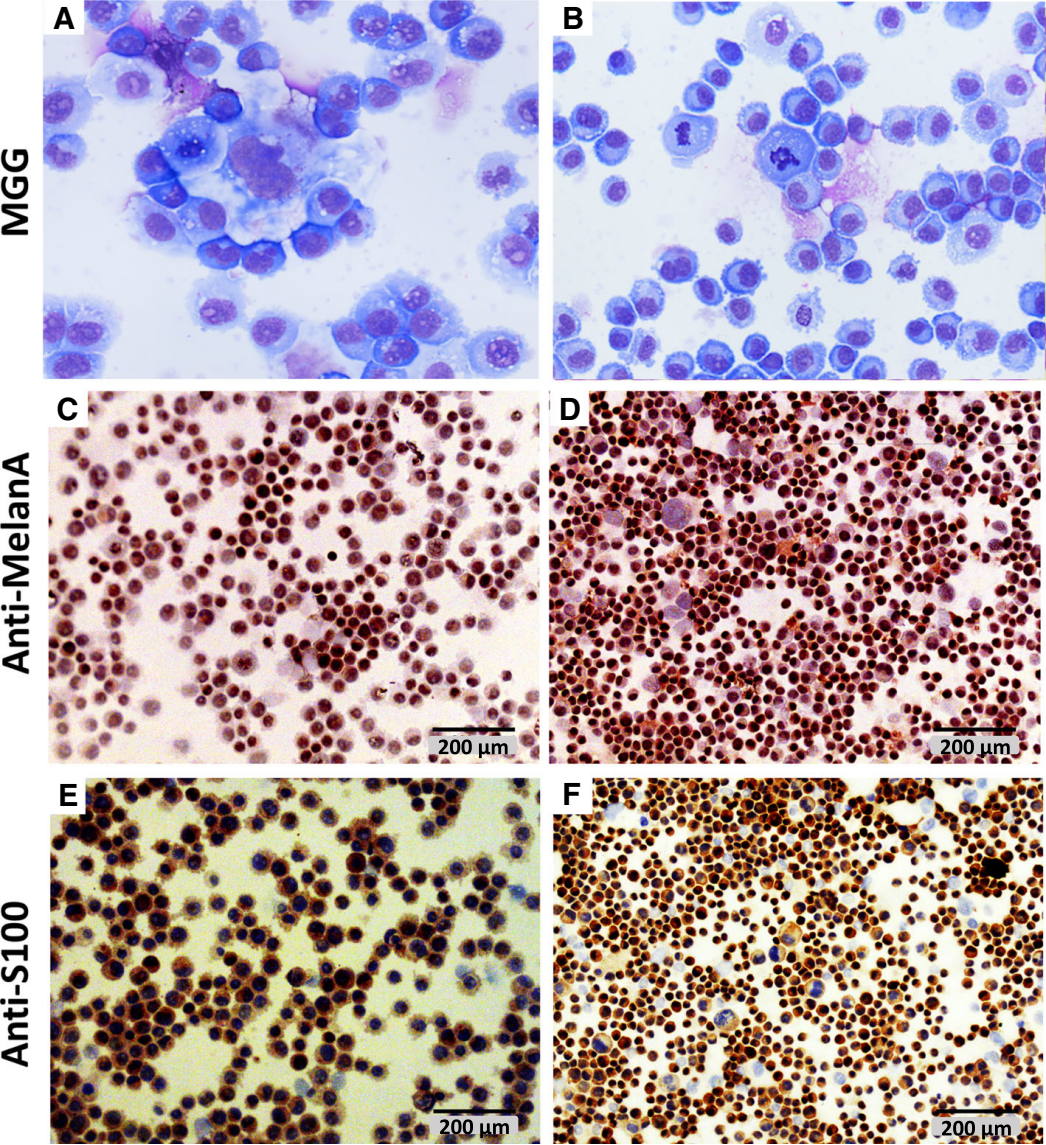
Immunocytochemical analysis of the isolated Ocr_OCMM1X (**a, c** and **e**) and Ocr_OCMM2X (**b, d** and **f**) cell lines. A large nucleus with disorganized, coarse chromatin and an elevated mitosis occurrence per high-power field (**a, b**) was observed. The cell marked strongly and at various levels Melan-A (**c, d**) and S100 (**e, f**) proteins. Observation under light microscope (magnification X40 for **a, b** and X200 for **c-f**)

These findings were confirmed by the evaluation of the cytoplasmic expression of Melan-A, which was found to be positive in the xenograft tumor samples (Fig. 6c, d) and the xenograft-derived isolated cell lines Ocr_OCMM1X and Ocr-OCMM2X (Fig. 7c, d and Additional file 4B). In addition, S100 protein expression was also found to be positive in the isolated cells, confirming the melanocytic origin of the established cell lines (Fig. 7e, f and Additional file 4B). The Sk-Mel28 human melanoma cell line was used as positive control (data not shown).

### Pharmacological characterization

The effects of dacarbazine, an alkylating agent used as chemotherapy in human metastatic melanoma, vemurafenib, a BRAF inhibitor, and LY294002, a PI3K inhibitor, were evaluated for both canine oral melanoma cell lines (Ocr_OCMM1X & Ocr_OCMM2X) and two human cutaneous melanoma cell lines as controls (A375 and Sk-Mel-28, both BRAF V600E-mutated). Dose-response curves and IC50 values were determined 72 h after treatment.

Dacarbazine has been used as the standard chemotherapy for metastatic melanoma since 1972. The rate of response to dacarbazine ranges from 5 to 15% [24].

**Fig. 8:**
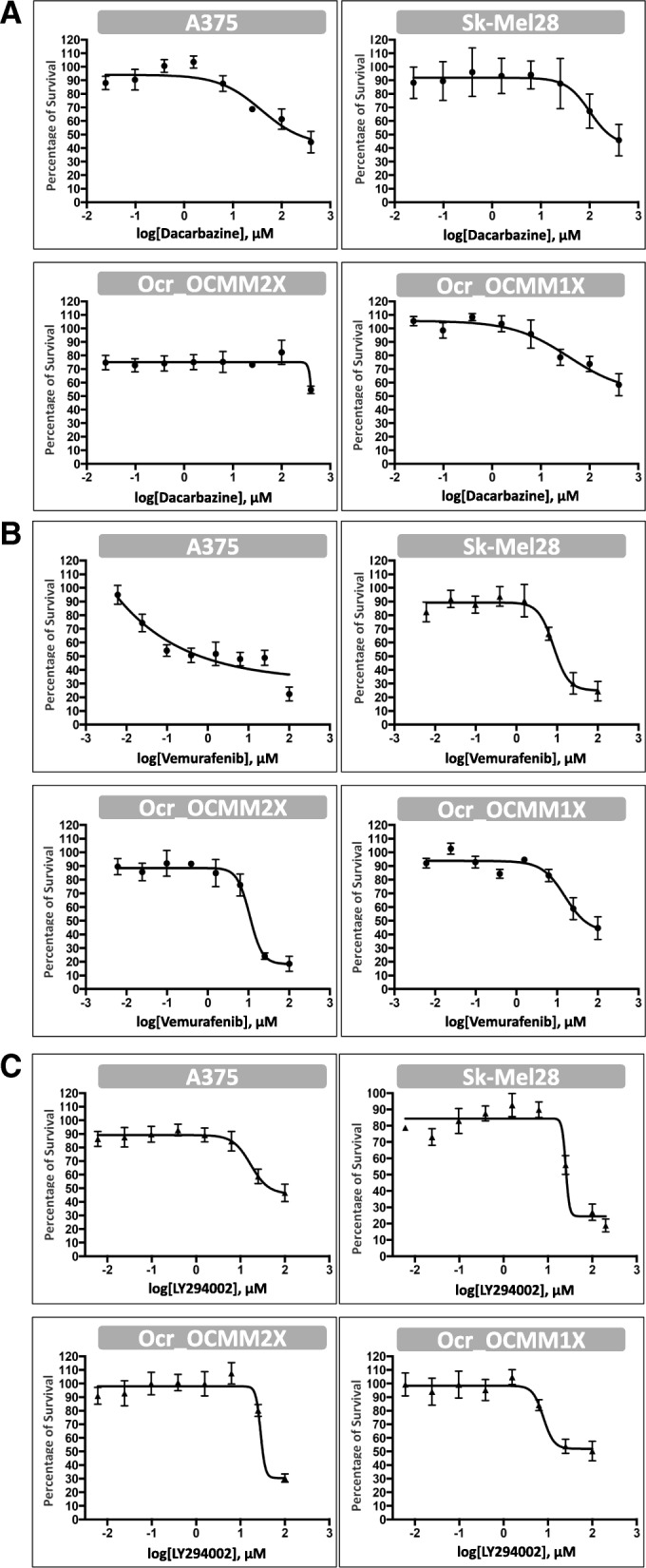
vHuman (A375 and Sk-Mel28) and canine melanoma cell lines (Ocr_OCMM2X and Ocr_OCMM1X) sensitivity to Dacarbazine (**a**), Vemurafenib (**b**) and LY294002 (**c**). Dose-response curves were generated after 72 h treatment with each compound and IC50 ± SEM values were determined using GraphPad Prism® software (*n* = 6)

The IC50 values for dacarbazine of the human cell lines were 202.4 ± 60.40 μM for A375 and 261.04 ± 31.55 μM for Sk-Mel-28, which are similar to values reported in the literature [25]. The IC50 values for dacarbazine were > 400 μM for both Ocr_OCMM2X and Ocr_OCMM1X cell lines (Fig. 8; Table 2).

**Table 2:** IC50 values of the isolated canine *and* human melanoma cell lines, 72 h after treatment with Dacarbazine, Vemurafenib and LY294002 (IC50 ± SEM, n = 6)

IC50 (μM)				
	A375	Sk-Mel28	Ocr_OCMM1X	Ocr_OCMM2X
Dacarbazine	202.4 ± 60,40	261.04 ± 31.55	> 400	> 400
Vemurafenib	0.54 ± 0.05	10.78 ± 3.29	37.44 ± 8.84	12.31 ± 0.85
LY294002	61.63 ± 16.85	26.56 ± 0.81	37.74 ± 9.6	32.39 ± 2.01

These values show a difference of response between the two cell-lines that might be explained by the more aggressive nature of the primary tumor higher stage of malignancy of a tumor collected from Dog_2 (TS2). Indeed, the Ocr_OCMM2X cell line originates from a highly metastatic melanoma, whereas Ocr_OCMM1X cells were obtained from a less aggressive tumor 32.39 ± 2.01 μM.

The IC50 values for vemurafenib of the human cell lines were 0.54 ± 0.05 μM for A375 and 10.78 ± 3.29 μM for Sk-Mel28, which are similar to the values reported in the literature [26]. The IC50 values for vemurafenib were 12.31 ± 0.85 μM and 37.44 ± 8.84 μM in canine cell lines Ocr_OCMM2X and Ocr_OCMM1X, respectively.

The IC50 values for LY294002 were 32.39 ± 2.01 μM and 37.74 ± 9.6 μM in canine cell lines Ocr_OCMM2X and Ocr_OCMM1X, respectively. Those values were slightly lower than the IC50 values of 61.63 ± 16.85 μM for A375 and higher than the IC50 values of 26.56 ± 0.81 μM for Sk-Mel28, which were similar to the values reported in the literature [27, 28] (Fig. 7; Table 2). IC75 values were also measure and are presented in Additional file 5.

These results show a difference of response between the two cell lines that could be explained by Dog_1 and Dog_2 tumors different stage of malignancy at the time of diagnosis and samples collection.

### MAPK and PI3K/Akt pathway activation

**Fig. 9:**
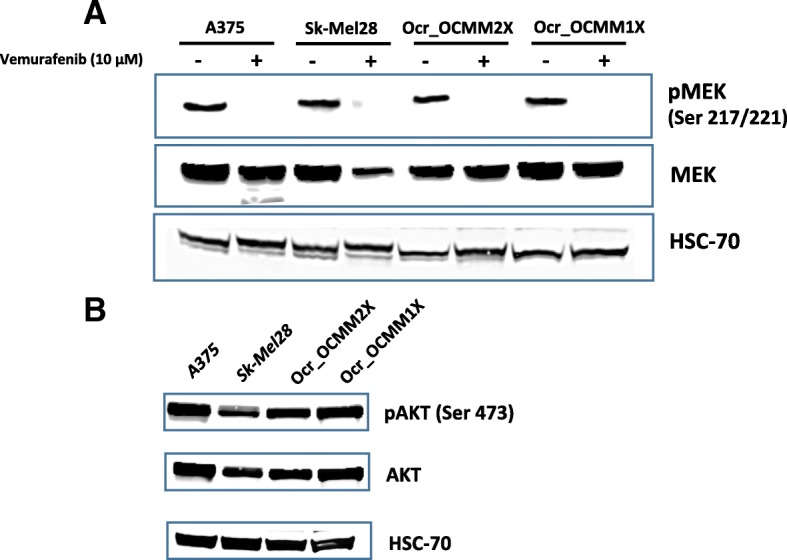
P-MEK, MEK (**a**) and pAKT, AKT (**b**) expression in the isolated canine melanoma cell lines (Ocr_OCMM2X and Ocr_OCMM1X). Cells were incubated for 24 h in the presence or absence of vemurafenib at 10 μM final concentration. A375 and Sk-Mel28 human cell lines were used as positive control while HSC-70 served as loading control

In this study, we showed a decrease in the phosphorylated isoforms of the MEK protein in Ocr_OCMM1X and Ocr_OCMM2X, which disappeared after 24 h incubation with 10 μM of vemurafenib (Fig. 9a). The presence of phosphorylated AKT protein was also shown, demonstrating that the PI3K/Akt pathway is activated in both xenograft-derived melanoma cell lines (Fig. 9b).

### Genetic analysis

All the samples were wild type for *Mitf* (E378), *Braf* (V595), *Gna11*, *Nras* (G12 and Q61), *Gnaq* and *p53*. However, no amplification of the 11 exons of TP53 was found in Dog_1-derived tumor and Ocr_OCMM1 cells, which was unexpected. We thus suspected a deletion of this region, which was further confirmed by CGH analysis.

### Genomic characterization

To order to identify the Copy Number Abbreviations (CNA) possibly involved in the tumorigenesis of these melanomas, we performed CGH analyses on the tumor DNA and derived cell lines of the 2 melanomas (see Additional files 6 and 7).

**Fig. 10:**
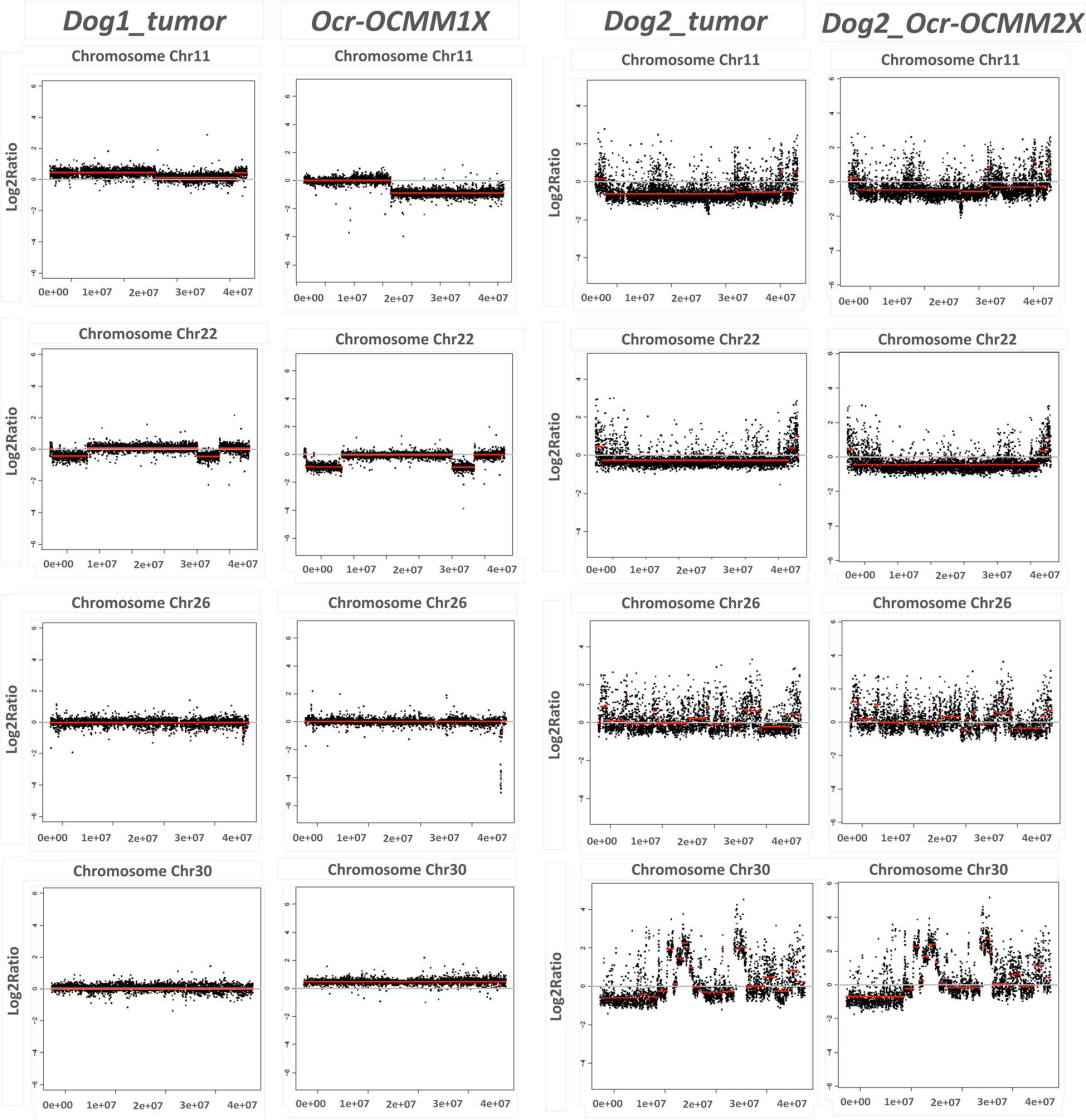
CGH profiles of canine chromosomes 11, 22, 26 and 30 in Dog_1 tumor, Ocr_OCMM1x, Dog_2 tumor and Ocr_OCMM2X. The diagrams were generated using a specific algorithm with R statistical computing software

The two tumors present distinctive genome-wide CGH profiles (see Additional files 6 and 7). The Dog_1 tumor is characterized by a few large CNAs involving large parts of the chromosomes, with 49 gains/losses (AMD2 segmentation method) vs.37 gains/losses using R scripts with an average size of 4.8 Mb, a SE of 23.2 Mb and a total size of 725 Mb. In contrast, the Dog_2 tumor is characterized by numerous focal alterations: 727 gains/losses with an average size of 0.82 Mb and a SE of 4.48 Mb. Among the altered regions, some CNAs had already been described as characteristic of and recurrent in oral melanomas in dogs [16] (Fig. 10). These CNAs included the loss of CFA22 within *RB1* (retinoblastoma tumor suppressor) gene in both tumors, and the loss of CFA26 within *PTEN* (phosphatase and tensin homologue) gene associated with the loss of a region of CFA11, including CDKN2A (cyclin-dependent kinase inhibitor 2A) in the Dog_2 tumor. Moreover, the Dog_2 tumor displayed a sigmoidal pattern of copy number loss followed immediately by a gain of CFA30, as already described in humans, specifically for oral melanomas.

Ocr_OCMM1X and Ocr_OCMM2X were obtained from these two tumors. The genome-wide CGH profiles reflect the clinical and severity status of each cell line (Fig. 10). Nearly 70.8% of the altered bases, observed gains or losses in the Dog_1 primary tumor and were found to be altered the same way in the corresponding cell line. Similarly, 77% of the CNAs observed in the Dog_2 tumor were also detected in Ocr_OCMM2X cell line, representing 92.7% of the nucleotides modified in the primary tumor. Moreover, among the altered regions, the CNAs that are characteristic of and recurrent in oral melanomas that had been detected in the primary cells were also detected in the cell lines (Fig. 10).

## Discussion

In this project, we described the characterization of two canine melanoma cell lines (Ocr_OCMM1X and Ocr_OCMM2X) that can be used as tools allowing the further validation and use of the canine model in the study of melanoma.

Only a few canine melanoma cell lines have already been reported in the literature. For instance, Inoue et al. established four canine melanoma cell lines (*CMec-1 CMec-2 KMec* and *LMec*) of various origins (subcutaneous, gingival and mucosal) from donors with different stages of development (T3 N1 M0 and T4 N1 M0). Of the three cases, no distant metastases were reported and only morphological characterization was performed [43]. Our study completed and endorsed this work by providing further validation of canine melanoma models for translational studies.

The isolated Ocr_OCMM1X and Ocr_OCMM2X cell lines reproduced the morphological, histological and copy number alteration features of the canine primary tumors from which they were derived. These cells were highly malignant and clearly showed potential for tumor formation in immunodeficient SHO Nude mice. Additionally, we were able to show the presence of melanoma-specific markers (Melan-A and S100) in both cell lines, confirming the melanocytic origin of these malignant cells.

The pharmacological sensitivity of the Ocr_OCMM1X and Ocr_OCMM2X cell lines to conventional chemotherapeutic agents has been evaluated. Continuous exposure to these agents led to the emergence of resistance in the Ocr_OCMM2X cell line, which correlated with the aggressiveness of the primary tumor. Dacarbazine constitutes the most widely used molecule in the treatment of melanomas, but the response rates associated with this drug are very poor. In fact, only 15–25% of treated human melanomas show signs of improvement with this agent [29, 30].

Several oncogenes are known to be linked to melanomagenesis, such as *Braf*, *Nras*, *Gna11*, *Gnaq*, *Mitf* and *p53.* Mutations in these genes are described in human cutaneous melanoma (BRAF, NRAS), mucosal melanoma (NRAS), blue nevi and uveal melanomas (GNAQ, GNA11) and in numerous other cancers (TP53) [17]. While more than 80% of human cutaneous melanomas are *BRAF*-mutated [31], this alteration is less frequent and poorly documented in dogs [32–34]. Although the mutational statuses of the A375 and Sk-Mel28 cell lines are well established, only 6% of canine melanomas harbor this alteration [17, 33, 34]. Despite their wild-type status with regard to this mutation, our cell lines responded positively to targeted therapies such as vemurafenib, a specific *BRAF* V600E mutation inhibitor, and we observed inhibited cell proliferation. According to *Tahiri* et al.*,* some melanoma tumors harboring wild-type BRAF could be sensitive to vemurafenib [35] (Fig. 8; Table 2).

Malignant melanoma is linked to several complicated signal transduction pathways that provide melanocytic malignant cell lines with the ability to proliferate, survive and invade tissues at remote sites. In addition to the mitogen-activated protein kinase (MAPK) cascade, the PI3K/Akt pathway constitutes an important signaling pathway in the progression, proliferation and survival of malignant melanocytes. To evaluate the extent of inhibition of the MAPK and PI3K/Akt pathways in our cell lines, Western blots were performed on native and phosphorylated MEK and AKT proteins.

The pharmacological results regarding vemurafenib inhibition were confirmed by Western blot analyses and showed an inhibition of the MAPK pathway and a significant decrease in the levels of phosphorylated MEK protein, as previously reported [36]. The results of the present study strongly suggest an inhibition of the MAPK pathway and a significant decrease in the levels of phosphorylated MEK protein.

Interestingly, the PI3K/Akt pathway was shown to be highly active in the Ocr_OCMM1X and Ocr_OCMM2X canine cell lines, confirming the importance of this pathway in melanomagenesis [37–39]. This activation could be explained by the upstream stimulation of kinase receptors such as c-Kit, which is known to be mutated in melanocytic tumors and confers on tumors the qualities of aggressiveness, disease progression and resistance to treatment [40].

Genetic profiling revealed different mutational landscapes of the two cell lines that were in accordance with the parent tumor profiles. Indeed, most CNAs observed in the primary tumors were detected in the related cell lines. The differences in the CNAs observed between primary tumors and the corresponding cell lines are potentially linked to the noise in the primary tumor due to the presence of wild-type non-tumorigenic cells but could also be the result of sub-clone selection during primary culture or cell line genetic drift though passages (Additional files 6 and 7). Nevertheless, the genome-wide CGH profiles of the derived cell lines reflected those of the tumors (Additional files 6 and 7), and interestingly, the cell lines present the characteristic CNAs of oral melanomas in dogs. In humans, alteration of the genes involved in cell cycle regulation or DNA repair, such as cyclin CDKN2A, p16^INK4A^ and PTEN, is known to be a major element driving the development of melanoma pathogenesis. These cytogenetic signatures have been identified in dog melanomas, including in our canine cell lines, and correlate with the potential aggressiveness of each parent tumor [16, 41]. In addition, the rearrangement in chromosome 30 reported in the Ocr_OCMM2X cell line correlates with the results of previous studies reported in the literature [16] and is consistent with the clinical behavior of the donor’s sample. *Poorman* et al. noted a remarkable similarity between canine melanoma genetic profiles and human orthologous variations with this complex copy number signature on CFA30/HSA15 [16]. Interestingly, this copy number signature is observed in the cell line derived from the metastatic tumor with chromosome instability (Dog_2 tumor), leading to the hypothesis that this genomic instability might be linked to the aggressiveness of the primary tumor (Additional file 7, Additional file 8 & Additional file 9).

## Conclusion

In conclusion, the results presented here endorse the relevance of canine models of spontaneously occurring diseases in the study of canine and human pathology. Due to the intact immune system present in dogs, the presence of a tumor niche and the fact that dogs share their environment with humans, canine oral melanomas are of great relevance to human diseases and represent a good opportunity for advancing researchers’ understanding of these rare tumors and for accelerating the translation of new findings to humans. In addition, because cytotoxic therapies are often associated with significant side effects and poorer pharmacodynamic activities, there is a strong need for new, effective, and less aggressive molecules. Extensive pharmacologic and genomic analyses of the presented canine cell lines now offer new tools for accelerating the translation of research findings between dogs and humans to achieve unified medicine and better patient care. Further studies are ongoing to isolate and identify dormant cancer stem cells that are potentially responsible for treatment failure and tumor relapses. Preliminary results show promising progress in the understanding of melanoma tumor biology and translatability to human clinical medicine.

## Additional files

Additional file 1: Summary of antibodies used for immunohistochemical and Western Blot analysis in the study. (TIF 77 kb)

Additional file 2: List of the primers used for gene sequencing. (TIF 362 kb)

Additional file 3: Immunohistochemical analysis of Dog_1 (**A, C**) and Dog_2 (**B, D**) engrafted tumors using Hematoxilin & Eosin (A, B) and Melan-A (C, D) (magnification X400). (PPTX 188530 kb)

Additional file 4: Immunofluorescent staining showing Melan-A and S100 protein expression in Ocr_OCMM1X (A, C) and Ocr_OCMM2X (B, D) cell lines. (PDF 242 kb)

Additional file 5: IC75 values of the isolated human (A375 and Sk-Mel28) and canine melanoma (Ocr_OCMM1X and Ocr_OCMM2X) cell lines, 72 h after treatment with Dacarbazine, Vemurafenib and LY294002. (TIFF 564 kb)

Additional file 6: CGH profiles of canine chromosomes 11, 22, 26 and 30 in Dog_1. Comparative analysis between the primitive tumor, xenograft tissue, Ocr_OCMM1X Passage 1 and Ocr_OCMM1X. The diagrams were generated using a specific algorithm with R statistical computing software. (PDF 2368 kb)

Additional file 7: CGH profiles of canine chromosomes 11, 22, 26 and 30 in Dog_2. Comparative analysis between the primitive tumor, Ocr_OCMM2 primary and Ocr_OCMM2X. The diagrams were generated using a specific algorithm with R statistical computing software. (PDF 3075 kb)

Additional file 8: Comparative analysis in CGH profiles of canine chromosomes 11, 22, 26 and 30 in Dog_2 vs. Dog_1 derived cells. Comparative analysis between Dog_2 primitive and xenograft derived tumors, Ocr_OCMM2 primary and Ocr_OCMM1X Passage 1. The diagrams were generated using a specific algorithm with R statistical computing software. (TIF 4433 kb)

Additional file 9: Comparative analysis in CGH profiles of canine chromosomes 11, 22, 26 and 30 in Dog_2 vs. Dog_1 derived cells. Comparative analysis between Dog_2 primitive and xenograft derived tumors, Ocr_OCMM2X and Ocr_OCMM1X derived cells. The diagrams were generated using a specific algorithm with R statistical computing software. (TIF 4864 kb)

## Abbreviations

ADM2: Aberration detection method 2
CDKN2A: Cyclin-dependent kinase inhibitor 2A
CGH: Comparative genome hybridization
CMM: Canine malignant melanomas
CNA: Copy number aberrations
GEO: Gene expression omnibus
HE: Haematoxylin-eosin
HRP: Horseradish peroxidase
MTS: (3-(4,5-dimethylthiazol-2-yl)-5-(3-carboxymethoxyphenyl)-2-(4-sulfophenyl)-2H-tetrazolium)
PDT: Population doubling times
PTEN: Phosphate and tensin homolog
RB1: Retinoblastoma tumor suppressor
s.c.: Subcutaneously and SEM: standard error of the mean
