# Supplementary figures and images for: Isolation and characterization of two canine melanoma cell lines: new models for comparative oncology
Source: BMC Cancer. 2018 Dec 4;18:1219. doi: 10.1186/s12885-018-5114-y (PMC6280433; doi:10.1186/s12885-018-5114-y)

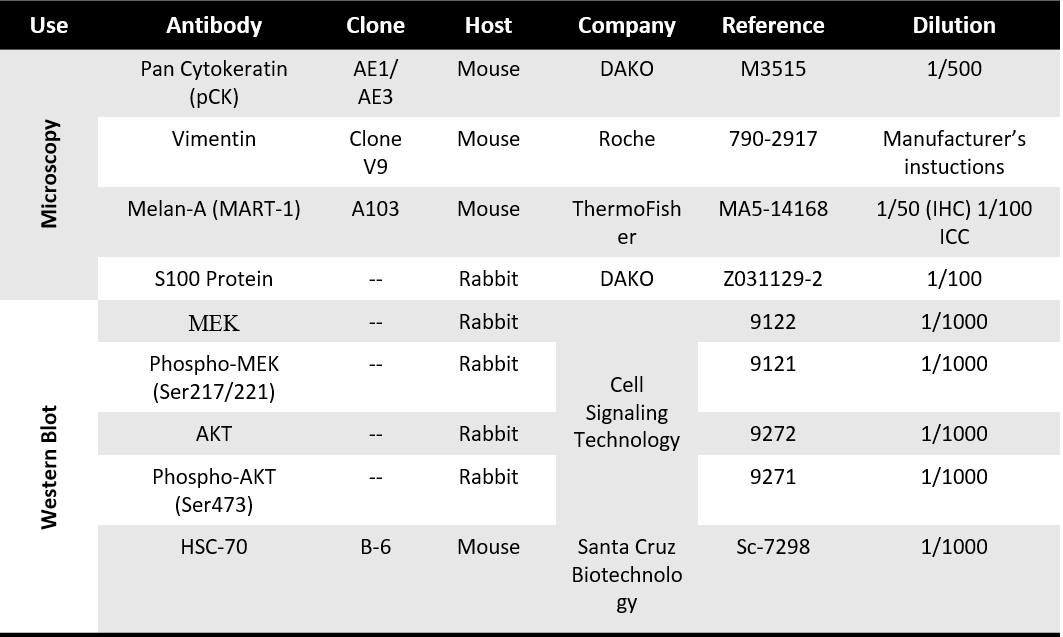

Supplement: Supplementary file 1 — Summary of antibodies used for immunohistochemical and Western Blot analysis in the study. (TIF 77 kb) [file 12885_2018_5114_MOESM1_ESM.tif]

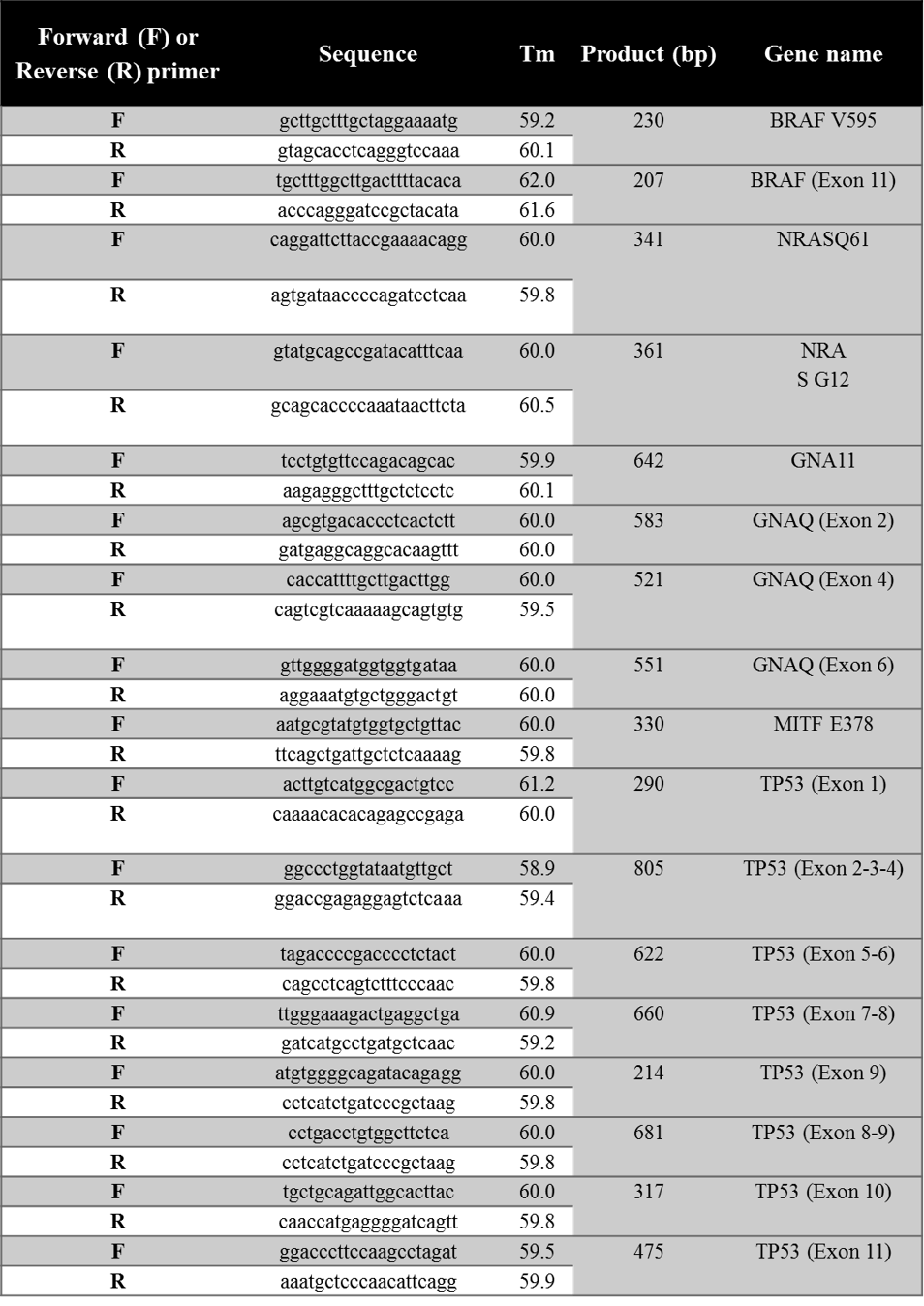

Supplement: Supplementary file 2 — List of the primers used for gene sequencing. (TIF 362 kb) [file 12885_2018_5114_MOESM2_ESM.tif]

Anti-MelanA

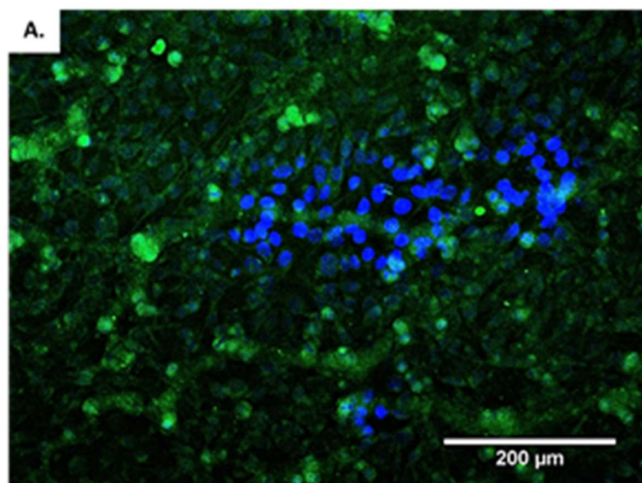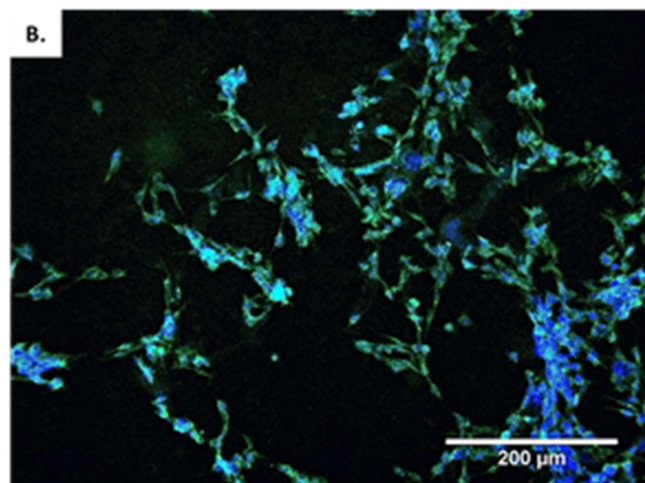

Anti-S100

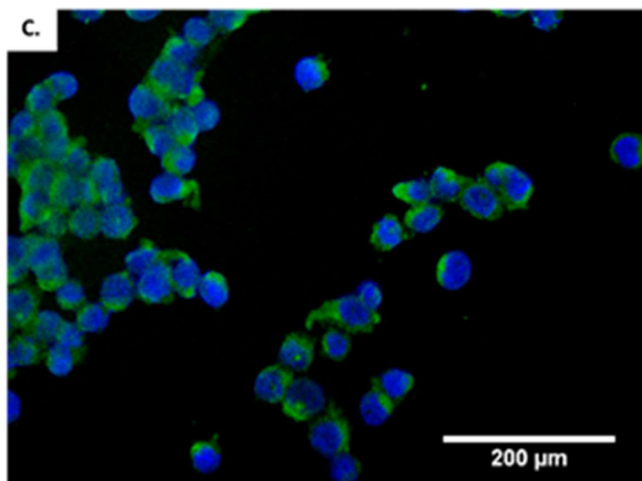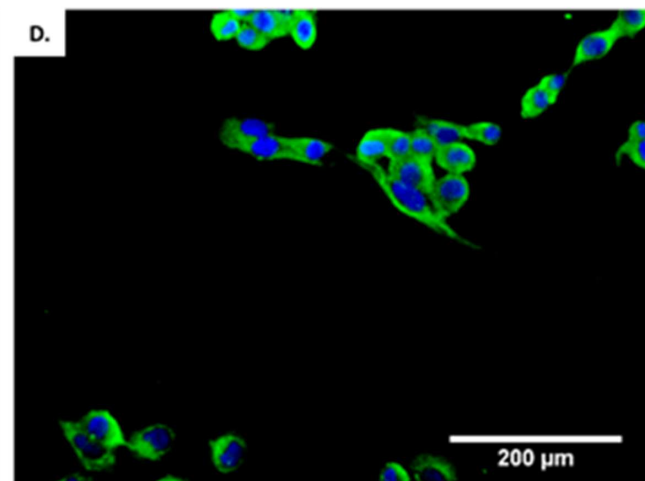

Supplement: Supplementary file 4 — Immunofluorescent staining showing Melan-A and S100 protein expression in Ocr_OCMM1X (A, C) and Ocr_OCMM2X (B, D) cell lines. (PDF 242 kb) [file 12885_2018_5114_MOESM4_ESM.pdf]

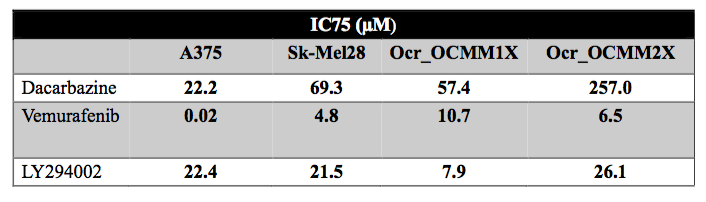

Supplement: Supplementary file 5 — IC75 values of the isolated human (A375 and Sk-Mel28) and canine melanoma (Ocr_OCMM1X and Ocr_OCMM2X) cell lines, 72 h after treatment with Dacarbazine, Vemurafenib and LY294002. (TIFF 564 kb) [file 12885_2018_5114_MOESM5_ESM.tiff]

Dog\_2

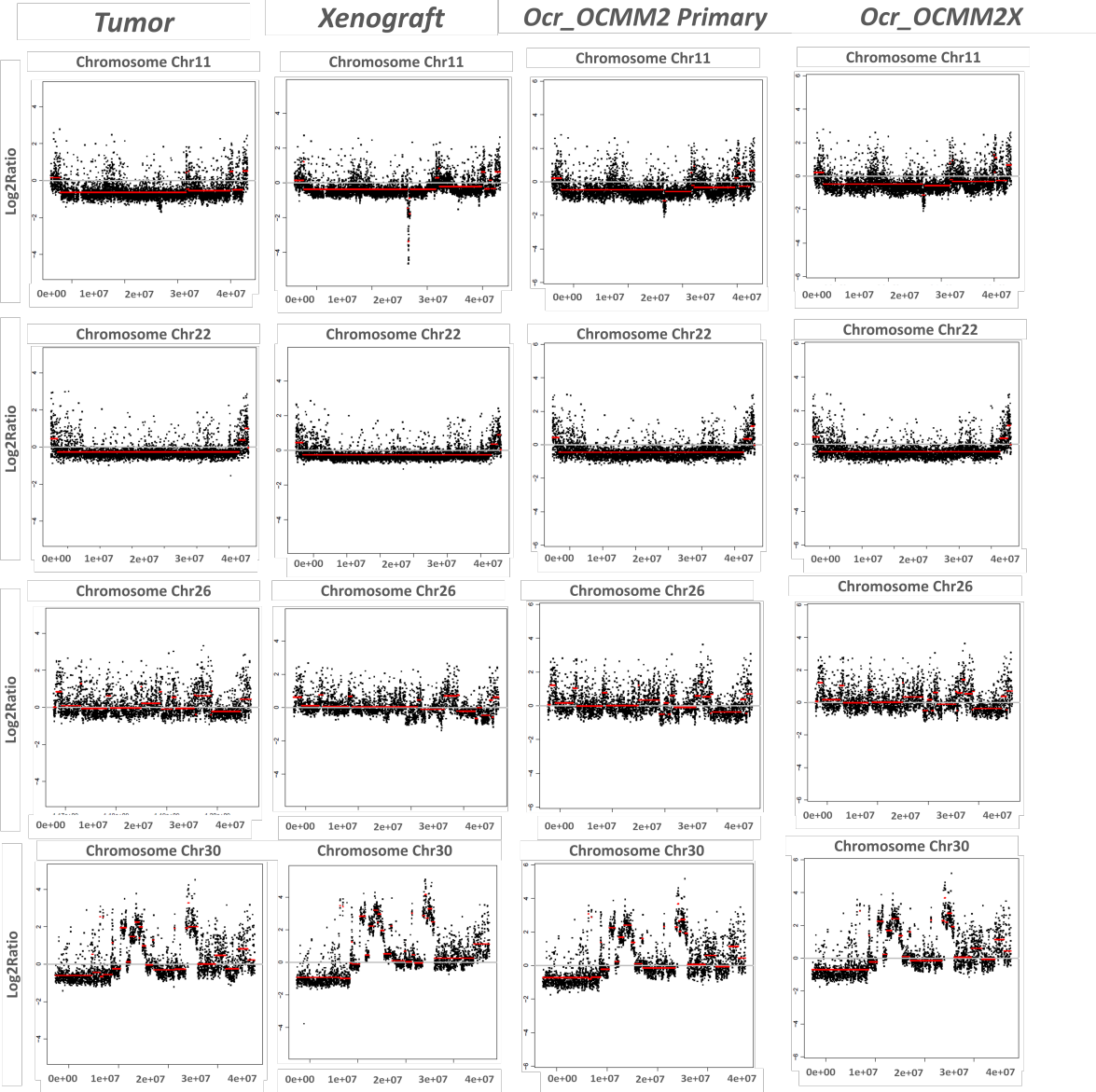

Supplement: Supplementary file 7 — CGH profiles of canine chromosomes 11, 22, 26 and 30 in Dog_2. Comparative analysis between the primitive tumor, Ocr_OCMM2 primary and Ocr_OCMM2X. The diagrams were generated using a specific algorithm with R statistical computing software. (PDF 3075 kb) [file 12885_2018_5114_MOESM7_ESM.pdf]

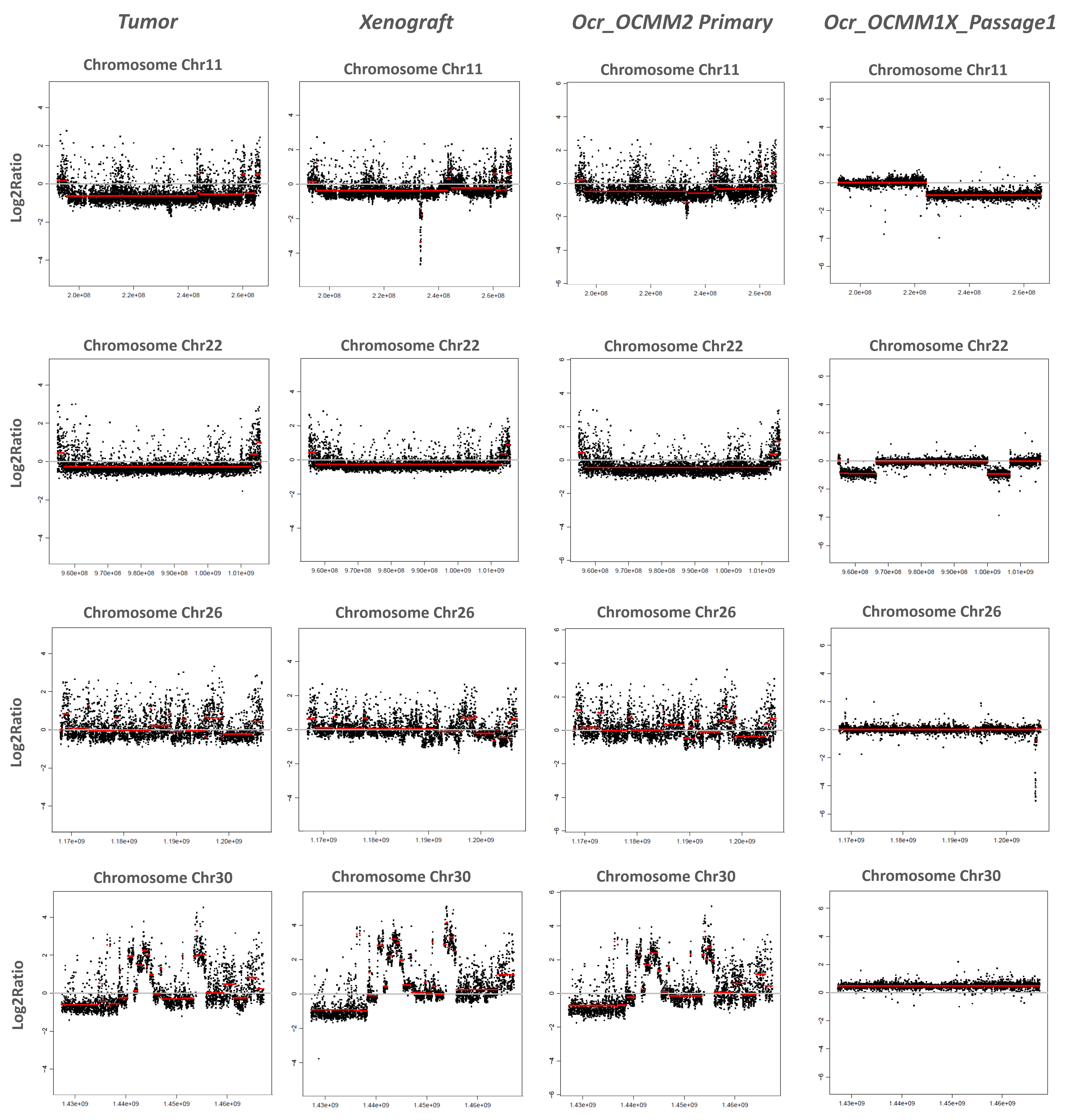

Supplement: Supplementary file 8 — Comparative analysis in CGH profiles of canine chromosomes 11, 22, 26 and 30 in Dog_2 vs. Dog_1 derived cells. Comparative analysis between Dog_2 primitive and xenograft derived tumors, Ocr_OCMM2 primary and Ocr_OCMM1X Passage 1. The diagrams were generated using a specific algorithm with R statistical computing software. (TIF 4433 kb) [file 12885_2018_5114_MOESM8_ESM.tif]

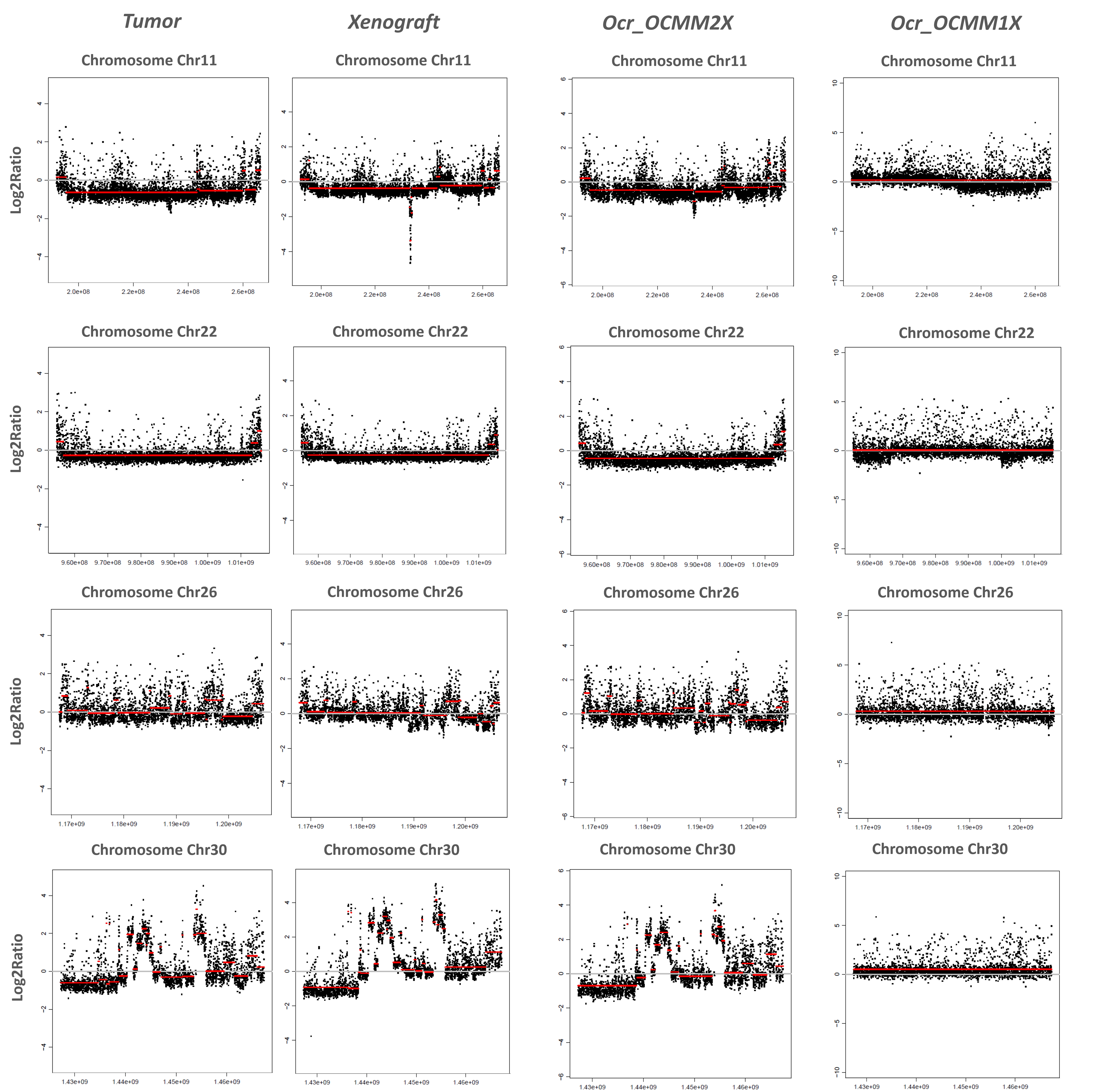

Supplement: Supplementary file 9 — Comparative analysis in CGH profiles of canine chromosomes 11, 22, 26 and 30 in Dog_2 vs. Dog_1 derived cells. Comparative analysis between Dog_2 primitive and xenograft derived tumors, Ocr_OCMM2X and Ocr_OCMM1X derived cells. The diagrams were generated using a specific algorithm with R statistical computing software. (TIF 4864 kb) [file 12885_2018_5114_MOESM9_ESM.tif]
