# Supplementary material for: Isolation and characterization of two canine melanoma cell lines: new models for comparative oncology
Source: BMC Cancer. 2018 Dec 4;18:1219. doi: 10.1186/s12885-018-5114-y (PMC6280433; doi:10.1186/s12885-018-5114-y)
Supplement: Supplementary file 6 — CGH profiles of canine chromosomes 11, 22, 26 and 30 in Dog_1. Comparative analysis between the primitive tumor, xenograft tissue, Ocr_OCMM1X Passage 1 and Ocr_OCMM1X. The diagrams were generated using a specific algorithm with R statistical computing software. (PDF 2368 kb) [file 12885_2018_5114_MOESM6_ESM.pdf]

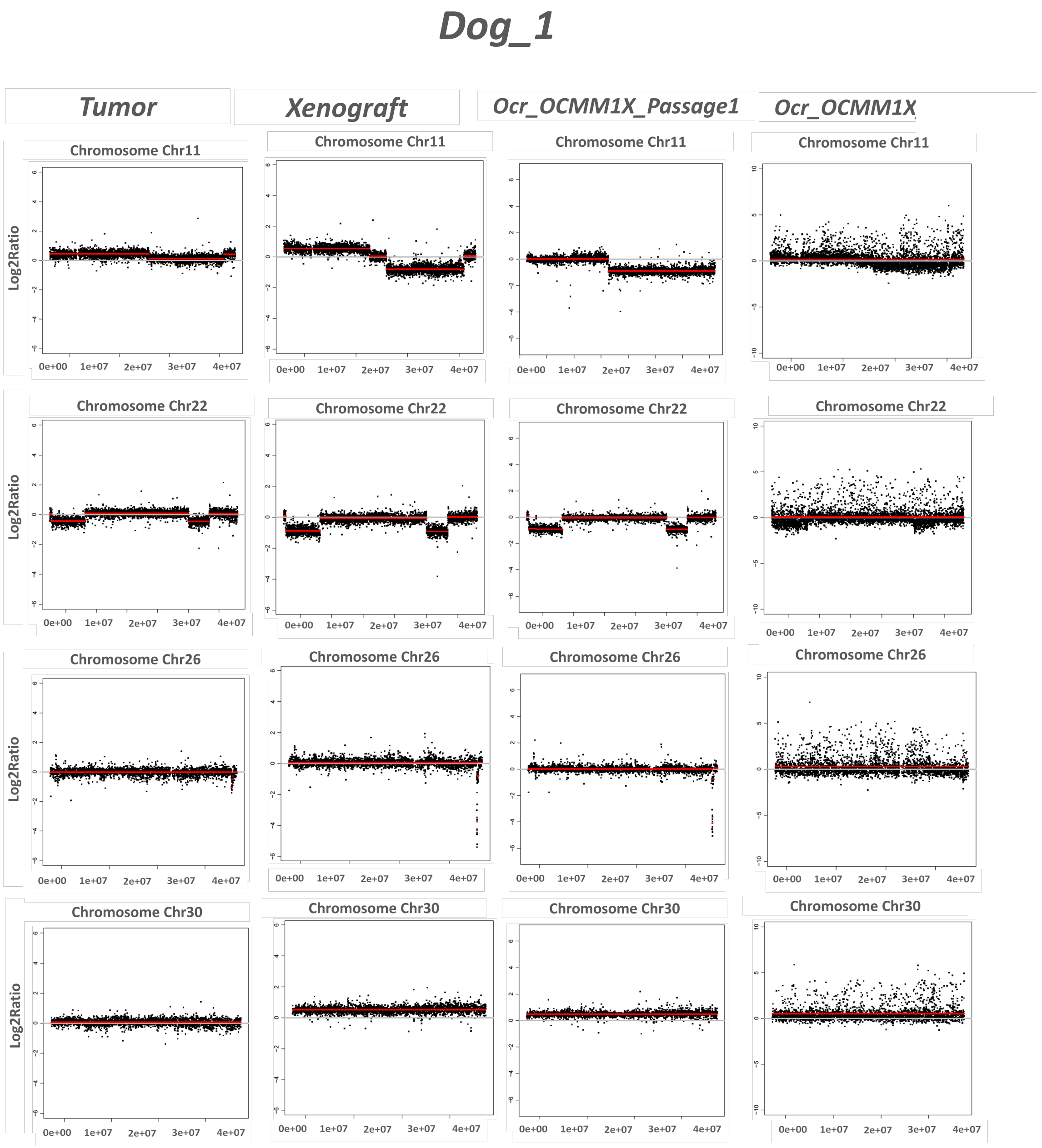

Figure S3a: CGH profiles of canine chromosomes (CFA) 11, 22, 26 and 30 in Dog_1. Comparative analysis between the primitive tumor, xenograft tissue, and derived cell lines. The diagrams were generated using a specific algorithm with R statistical computing software.
